# Atrioventricular thrombus in a 14-year-old patient: a case report

**DOI:** 10.1186/1757-1626-3-46

**Published:** 2010-02-02

**Authors:** Yavuz Besogul, Fatih Yılmaz, Birsen Uçar, Zubeyir Kılıç

**Affiliations:** 1 Department of Cardiovascular Surgery, No 67/22 Alpata Evleri, Eskisehir, Turkey; 2 Department of Pediatric Cardiology, Osmangazi University Medical School and Research Hospital, Eskisehir, Turkey

## Abstract

Right atrioventricular thrombus was diagnosed by echocardiography in a 14-year-old boy. Thrombus was reached through the right ventricle to the pulmonary artery and it was caused to tricuspit valve insufficiency. Surgical thrombectomy was performed and, he was treated with oral anticoagulation in postoperative period.

## Introduction

Atrioventricular thrombus are relatively rare in the pediatric population, but when present they are a potential source of significant morbidity and mortality[1]. Echocardiography is a widely accepted method to detect intracardiac thrombosis. The echocardiographic appearance of intracardiac thrombus is a mainly apical mass with a defined border to cavum and myocardium [2-5] The treatment of intracardiac thrombus has been surgical thrombectomy or thrombolytic agents such as tissue plasminogen activator, streptokinase, or urokinase as well as anticoagulants such as heparin or warfarin.

## Case Report

**Figure 1:**
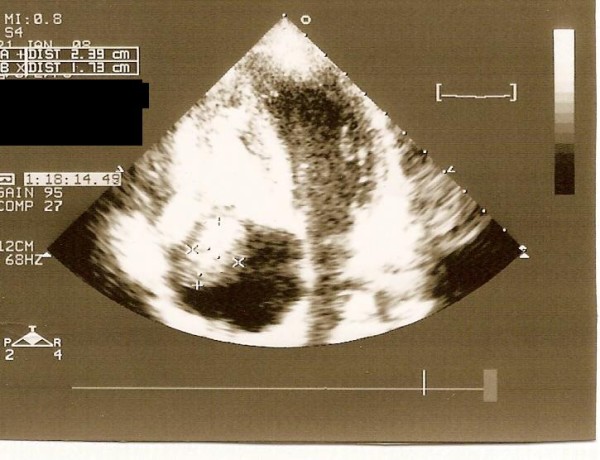
**Echocardiographic view of the thrombi in the right atrium**.

**Figure 2:**
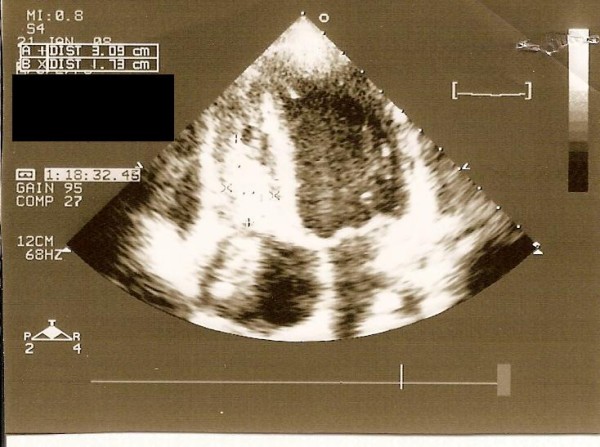
**Echocardiographic appearance of thrombi in the right ventricle**.

**Figure 3:**
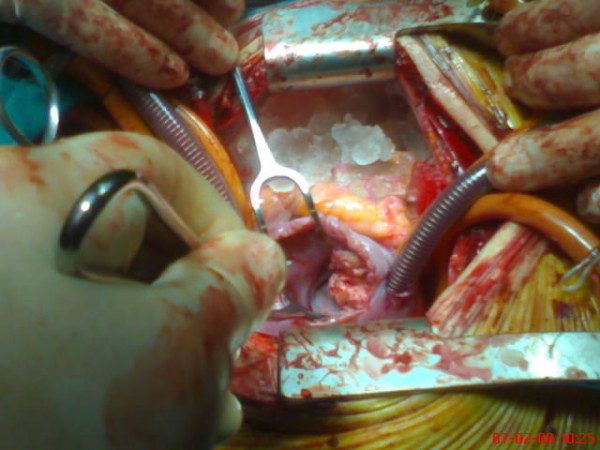
**The appearance of thrombus from right atrium in the operation**.

**Figure 4:**
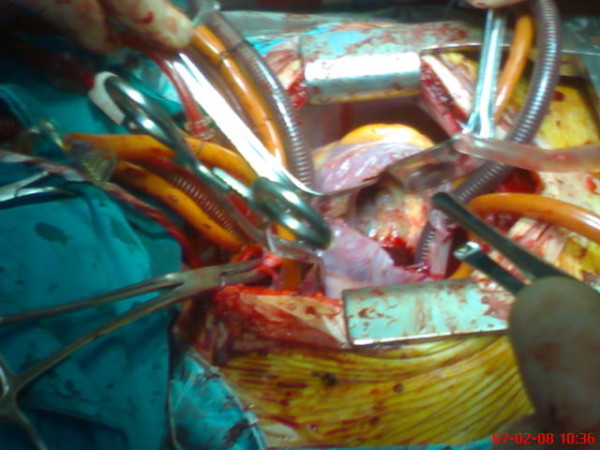
**Operative photograph of the thrombus from the right atrium to the right ventricle**.

**Figure 5:**
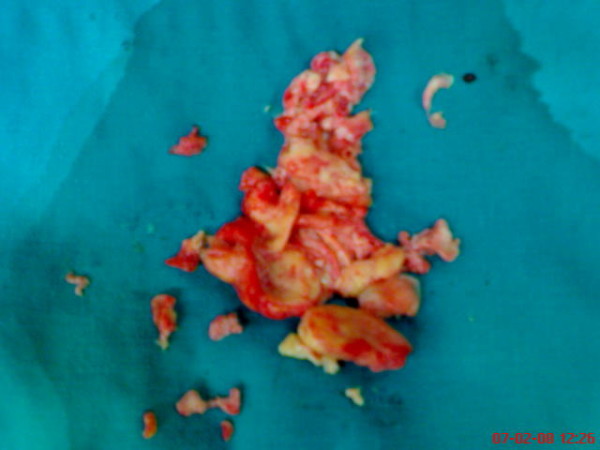
**The appearance of thrombus after thrombectomy**.

A 14-year-old male who had suffered from chill, nausea and vomiting for two weeks, was admitted to our hospital. Chest x-ray film and routine biochemical screening including thyroid status were normal, and the ECG was a sinus rhythm. An infectious or autoimmunologic disease was not evident by immunologic and serologic tests. Cross-sectional echocardiography and transesophageal echocardiography demonstrated that there were masses which were 33 × 26 mm in the right atrium and 26 × 22 mm in the right ventricle (Figure 1, 2). There was no atrial septal defect. In the cardiac magnetic resonance (MR), these mass were thrombus and continued to pulmonary artery. It was decided to carry out surgical thrombectomy. Once, a median sternotomy and pericardiatomy, heparine (300 Units/kg) was administered intravenously before cannulation for cardiopulmonary bypass(CPB) and additional dose was given to maintain an activated clotting time of 450 or faster. Arterial cannulation was performed via the aortic root and venous return was supplied by direct superior vena caval cannulation. Cross clamping was placed to aorta when moderate hypothermia (32°C) was occured and cardioplegia was infused into the aortic root for myocardial protection. When the right atrium was opened, the thrombus was appeared in the atrium through the ventricle via tricuspit valve. It was reached to pulmonary artery (Figure 3, 4). Therefore, the pulmonary artery was clamped. When the atrioventricular thrombus was removed (Figure 5), the tricuspit was evaluated and found to be insufficiency. The atrium and ventricular wall was observed granular structure. After the valve was repaired, atrium was closed with a double layer of 3/0 propylene sutures. The cross clamp and pulmonary clamp were removed and cardiac rhythm was spontaneously begun. Decannulation and neutralization of heparin were performed and, sternum was closed as usual. Postoperative echocardiography showed that there was no thrombus. The patient had an uneventful postoperative course with normal sinus rhythm and he was discharged from the hospital symtom-free with oral anticoagulation.

## Discussion

Intracardiac thrombus, although relatively rare in the pediatric age group, have been increasing in recent years [6-8]. Pediatric intracardiac thrombus are most commonly diagnosed in patients with dilated cardiomyopathy and in patients status post Fontan operation. There is a male predominance[1]. The etiology in pediatric patients has yet to be clearly defined. Most pediatric studies have focused on underlying hematologic abnormalities, such as deficiencies in protein C and Factor V, that could predispose one to have an intracardiac thrombus [9,10]. Right heart thrombus occurs in association with the presence of central venous catheters, vegetations due to endocarditis, polycytaemia, congenital heart defects, respiratory distress syndrome and persistent foetal circulation [11,12]. They have been shown to cause superior vena cava syndrome, occlusion of the tricuspit valve, and valvuler insufficiency with rapidly progressive heart failure. Additionally, main risk of the patients consists in unpredictable pulmonary embolization[13]. Ebato et al[14], reported that the patients have presented with symptoms of cerebral embolism and pulmonary embolism. The traditional therapy for intracardiac thrombus in pediatric patients has been surgical thrombectomy. In the largest pediatric study to date Ozkutlu et al[6], described the outcomes of 11 patients with intracardiac thrombus, 5 of whom underwent thrombectomy. Thrombolytics such as tissue plasminogen activator, streptokinase, or urokinase have been used routinely for the treatment of neonatal thrombus [15,16]. The optimal treatment modalities for children with intracardiac thrombus are not known. Information on management is limited to case reports and small case series. Moreover, spontaneous regression of intracardiac thrombus has been previously reported[13]. There was a tendency for smaller cardiac thrombus to resolve with medical therapy and for embolization to occur in patients with large thrombi[1]. We have performed surgical embolectomy because of the large thrombus in the atrium and ventricle.

## Competing interests

The authors declare that they have no competing interests.

## Authors' contributions

YB; surgical treatment, study design, data analysis and writing

FY; surgical photographs, and literature collection

BU; diagnosis, echocardiography and preparation of the patient

ZK; diagnosis and echocardiography

All authors have read and approved the final manuscript.

## Consent

Written informed consent was obtained from the patient for publication of this case report and accompanying images. A copy of the written consent is available for review by the Editor-in-Chief of this journal.
